# Molecular analyses of unselected head and neck cancer cases demonstrates that human papillomavirus transcriptional activity is positively associated with survival and prognosis

**DOI:** 10.1186/s12885-016-2398-7

**Published:** 2016-06-13

**Authors:** Liam Masterson, David M. Winder, Siolian L. R. Ball, Katie Vaughan, Martin Lehmann, Lars-Uwe Scholtz, Jane C. Sterling, Holger H. Sudhoff, Peter K. C. Goon

**Affiliations:** Department of Pathology, University of Cambridge, Cambridge, UK; Department of Otorhinolaryngology, Cambridge University Hospitals NHS Trust, Cambridge, UK; Department of Otorhinolaryngology, Bielefeld Academic Teaching Hospital, Bielefeld, Germany

**Keywords:** Human papillomavirus, HNSCC, p53, Molecular diagnosis and prognosis

## Abstract

**Background:**

Human papillomavirus DNA detection in head and neck squamous cell carcinoma has been linked to improved patient prognosis. The main aims of the study was to test the hypotheses that HPV16 E6/E7 oncogene and p53 function within tumours were associated with the widely reported improved patient survival and prognosis in head and neck cancer.

**Methods:**

HPV16 DNA, mRNA and p53 mRNA presence were analysed in a prospective study of 42 unselected HNSCC patients; correlating the data with patient age, tumour staging/grade, treatment response, disease recurrence and survival.

**Results:**

HPV16 DNA and HPV16 mRNA were present in 45.2 % and 21.4 % of patients, respectively. There was a significant positive association between the detection of HPV16 E6/E7 mRNA and p53 mRNA (*p* = 0.032), but this was not replicated for HPV16 DNA. Five-year disease free survival for the whole cohort was 63 % (CI 52.5–73.5 %). Multivariable analysis revealed only HPV16 E6/E7 mRNA expression to have significant prognostic influence (*p* = 0.04).

**Conclusions:**

Our study suggests that HPV16 oncogenic transcriptional activity within HNSCC tumours is associated with improved patient survival and better prognosis in a German population. Simple HPV DNA detection alone did not demonstrate this association. The significant association of full-length (wild-type) p53 with HPV16 E6/E7 mRNA is further evidence for a functional relationship, which could contribute to the widely reported improved survival and prognosis. Larger studies are required to validate the frequency of HPV16 mRNA expression in HNSCC.

## Background

Head and neck squamous cell carcinoma (HNSCC), with a worldwide incidence rate of approximately 500,000 new diagnoses per annum, is the 6^th^ most common form of cancer [1]. Mortality has not improved substantially over the last few decades [2], due to late diagnosis (75 % of cases) and/or recurrent primary malignancies, and remains at 40–50 % at 5 years [3, 4]. Risk factors for the development of HNSCCs include tobacco and alcohol use as well as oral human papillomavirus (HPV) infection [5].

Human papillomavirus (HPV) has an important pathogenic role for a subset of HNSCC [5–8], leading to the grouping of oropharyngeal tumours into HPV-negative and HPV-positive cancers. Meta-analyses have been used to assess the potential worldwide prevalence of HPV in HNSCC, resulting in an estimate of 26–35.4 % [7, 8]. A recent systematic review of oropharyngeal subsite cancers and HPV prevalence found a significant increase when comparing studies recruiting patients before 2000 and after 2005 respectively (40.5 % [CI 35.1–46.1 %] versus 72.2 % [CI 52.9–85.7]) [9].

HPV status of HNSCC has been shown to affect overall patient prognosis, HPV-positive tumours have an improved response to therapy as reflected in a significant reduction in the risk of progression and improvement in overall survival rate compared to HPV-negative malignancies, irrespective of the treatment regime used [10–13]. However, a certain number of patients with HPV-positive tumours have similar clinical outcomes to those seen in HPV-negative disease, the latter being linked to other prognostic factors such as increased EGFR expression [14] or smoking status [15].

As the fraction of HPV-positive head and neck cancers increases, this may highlight the importance of understanding the underlying mechanisms responsible for oncogenesis and the differences between HPV-related and HPV-unrelated disease that determine clinical outcome [16, 17].

The detection of HPV DNA alone, in the absence of evidence for viral gene expression, is not unequivocal molecular evidence that HPV infection either causes or promotes malignant progression in a lesion. In order to address this point, we have used a highly sensitive PCR detection method, together with a commercially available HPV genotyping kit (Linear Array HPV genotyping test, Roche Diagnostics Ltd., UK), to determine the presence of high- and low-risk HPV subtype L1 DNA in HNSCC tumours. Furthermore, we employed a separate PCR assay to detect HPV16 E6/E7 DNA and mRNA expression [18]. The ability of HPV 16 E6 to bind and promote the degradation of p53 has been suggested as one reason for an absence of disruptive p53 mutations in HPV 16 positive HNSCCs [19]. Full-length p53 RNA expression was therefore examined in age-matched groups of HPV16 E6/E7 expressing and non-expressing tumours. Patient data was collected, including patient age, sex, tumour staging, treatment response, recurrence and mortality. The relationship between the detection of HPV DNA, RNA, p53 expression and clinical data was then analysed. To further investigate discordant results, subgroup analysis was undertaken utilizing p16^INK4a^ immunohistochemistry.

Although p16^INK4A^ is the most commonly used biomarker for HPV positive head and neck cancer, specific limitations need to be observed. There is a subgroup of HPV- HNSCCs where expression of p16^INK4A^ could lead to erroneous classification as HPV positive. Immunohistochemical staining itself can produce false positives i.e. intra-observer/inter-observer variation of background stain. In addition, a small but significant cohort of the false positive HPV- samples have p16^INK4A^ mutations that may account for accumulation of inactive p16^INK4A^ [15].

## Methods

### Patients and specimens

Clinical samples were obtained from unselected patients attending the Bielefeld Academic Teaching Hospital, Department of Otorhinolaryngology, Bielefeld, Germany between 2008 and 2009. All patients gave written informed consent for the study, which was approved by the local research ethics committee (Ethik-Kommission der Ärztekammer Westfalen-Lippe). A sample of the tumour was taken for histological analysis and the remainder was snap-frozen in liquid nitrogen and transported to the UK on dry ice prior to DNA and RNA extraction. A consultant histopathologist with expertise in head and neck pathology reviewed each tissue block to ensure adequate tumor sampling. All experiments were performed in the Department of Pathology at the University of Cambridge.

The study comprised 42 HNSCC tissue samples (patient mean age = 62.9 years, 95 % CI 59.11–66.01, range 46–87). Tumour staging was classified using the TNM classification of malignant tumours [20]. Post-operative follow-up was conducted at regular intervals for a period of five years, assessing response to treatment, disease recurrence and patient mortality. Clinical and histopathological features of the 42 HNSCC patients are shown in Table 1.

**Table 1:** Clinical, histopathological and follow-up data for 42 HNSCC patients

Variable		Number	Percent
Site	Larynx	16	38.1
	Oropharynx	15	35.7
	Hypopharynx	5	11.9
	Oral cavity	4	9.5
	Other^b^	2	4.8
T stage^a^	1	8	19.0
	2	10	23.8
	3	10	23.8
	4	13	30.1
	n/a	1	2.4
N stage^a^	0	17	40.5
	1	6	14.3
	2	17	40.5
	3	1	2.4
	n/a	1	2.4
M stage^a^	0	40	92.9
	1	0	4.8
	X	2	2.4
Grade	Well	0	0
	Mod	27	64.3
	Poor	12	28.6
	n/a	3	7.1
	Surgery	27	64.2
Treatment	RT	13	30.9
	CRT	2	4.7
Death	Yes	9	21.4
	No	33	78.6
Response^c^	Complete	29	69.0
	Partial	4	9.5
	None	7	16.7
	n/a	2	4.8
Recurrence	Yes	13	30.9
	No	29	69.1

T represents size or direct extent of the tumour, N the degree of lymph node spread and M the presence of metastases

^a^Pathological TNM staging; ^b^Two patients had primary SCC located in EAC; RT, primary radiotherapy; CRT, primary chemoradiotherapy; ^c^Post-treatment clinical evaluation was undertaken at ~8-12 weeks [complete response is defined as the disappearance of all detectable disease at the primary site on visual inspection and/or imaging; partial response was defined as tumour reduction by >50 %]

### DNA extraction

Samples (max. 25 mg) were disrupted in a Bullet Blender™ (Next Advance, Averill Park, USA) in 300 μl digestion mix (10 mM Tris, pH 7.5; 10 mM EDTA; 0.5 % SDS; 200 μg/ml Proteinase K) for 5 min and then incubated overnight at 37 °C. Following Proteinase K inactivation at 56 °C for 10 min, the lysate was subjected to a phenol:chloroform extraction (1:1:1 volume) and the DNA precipitated from the supernatant with 1 ml 100 % ethanol. The DNA was then centrifuged (13,000 rpm, 4 °C, 20 min), the pellet washed with 70 % ethanol, air dried and re-suspended in 200 μl PBS. RNase digestion and total genomic DNA isolation was then performed using the DNeasy Blood and Tissue Kit (QIAGEN Ltd, UK), according to the manufacturer’s instructions. DNA was eluted with purified (deionised, double-distilled) H_2_O, and after purity and concentration were ascertained using a Nanodrop™ 1000 spectrophotometer, stored at −20 °C until use.

### RNA extraction and cDNA synthesis

Samples (max. 25 mg) were disrupted in a Bullet Blender™ (Next Advance, Averill Park, USA) in 400 μl TRIzol reagent (Invitrogen Ltd, UK) for 5 min. A further 600 μl of TRIzol was added to each sample and the lysate subjected to a chloroform extraction (200 μl). The supernatant was precipitated with 0.5 ml isopropanol o/n at −20 °C. The RNA was then centrifuged (13,000 rpm, 4 °C, 20 min), the pellet washed with 70 % ethanol, air dried and re-suspended in 50 μl H_2_O. DNase I digestion (Invitrogen Ltd, UK) was then performed prior to RNA clean-up using a PureLink™ RNA Mini Kit (Invitrogen Ltd, UK), according to the manufacturer’s instructions. RNA was eluted with purified (deionised, double-distilled) H_2_O, purity and concentration was ascertained using a Nanodrop™ 1000 spectrophotometer.

Up to 5 μg of total RNA was reverse transcribed using BioScript Reverse Transcriptase (Bioline Ltd, London, UK) according to the manufacturer’s instructions. Briefly, RNA was incubated with random primers (Invitrogen Ltd, UK) at 70 °C for 5 min before being placed on ice for 5 min. Following the addition of reaction buffer, dNTPs and reverse transcriptase, cDNA synthesis was performed under the following conditions: 25 °C for 5 min, 42 °C for 30 min (synthesis step) and 70 °C for 10 min (stops reaction). Samples were stored at −20 °C prior to use. Reverse transcriptase negative samples acted as negative controls for subsequent analyses.

## PCR methods

### L1 single step DNA PCR analysis

A PCR assay using the PGMY09/11 L1 consensus primer set was performed as described previously [21]. The PCR cycling conditions were as follows; denaturing step of 95 °C for 5 min, followed by 40 cycles of 95 °C for 1 min, 55 °C for 1 min and 72 °C for 1 min. This was followed by a final extension period of 10 min at 72 °C. Agarose gel electrophoresis was used to confirm the presence/absence of bands specific for both HPV and human β-globin. Human placental DNA (Sigma-Aldrich, UK), containing 1000 copies of the plasmid pSP64-HPV16 [22], was used as a positive control for this and subsequent HPV16 PCRs of genomic DNA.

### L1 nested DNA PCR and direct cycle sequencing

PCR reactions that were negative following amplification with the PGMY09/11 L1 consensus primers were subjected to further PCR amplification using the GP5+/GP6+ primer pair as described previously [23]. The PCR cycling conditions were as follows; denaturing step of 95 °C for 5 min, followed by 30 cycles of 95 °C for 1 min, 40 °C for 2 min and 72 °C for 1.5 min. This was followed by a final extension period of 10 min at 72 °C.

Positive bands identified following agarose gel electrophoresis were excised, the DNA purified using QiaQuick Gel Extraction columns according to the manufacturer’s instructions (QIAGEN Ltd, UK) and sequenced directly (Geneservice Ltd, UK) using the GP5+/GP6+ primers. The sequences were then aligned with known HPV types (NCBI Basic Local Alignment Search Tool).

### PGMY-line blot assay/Linear Array HPV genotyping test

The procedure was carried out according to the manufacturer’s instructions and as previously described [24]. Briefly, PCR amplification was carried out with LA HPV GT primers as provided: Each 100 μl reaction consisted of 50 μl working master mix containing MgCl_2_, KCl, Amplitaq Gold DNA polymerase, uracil-N-glycosilase, deoxynucleotides (dNTPs) and biotinylated PGMY and β-globin primers together with 50 μl of DNA sample. DNA templates were titrated to a concentration of 2–4 ng/μl, i.e. 100–200 ng template DNA per reaction. The Applied Biosystems gold-plated 96-Well GeneAmp PCR System 9700 was programmed as follows: 50 °C for 2 min, 95 °C for 9 min and 40 cycles of 95 °C for 30 s, 55 °C for 1 min, 72 °C for 1 min and finally, at 72 °C for 5 min before holding indefinitely at 72 °C. The 40 cycles had a ramp rate set at 50 %.

The amplicons were denatured and hybridised to a strip containing specific probes for 37 HPV genotypes and β-globin reference lines before undergoing stringent washes.

Colorimetric determination with a Linear Array Detection Kit: the colour change reaction was from streptavidin-horseradish peroxidase mediated precipitation of working substrate. Positive reactions appeared as blue lines on the strip. The strips were interpreted using the HPV reference guide provided.

### E6/E7 DNA PCR analysis

A PCR assay using primers specific for HPV16 E6/E7 was performed as described previously [25]. The PCR cycling conditions were as follows; denaturing step of 94 °C for 5 min, followed by 40 cycles of 94 °C for 1 min, 60 °C for 45 s and 72 °C for 1 min. This was followed by a final extension period of 10 min at 72 °C.

### E6/E7 cDNA PCR analysis

cDNA generated from extracted HNSCC mRNA was subjected to E6/E7 PCR analysis using the same primers and conditions as used for DNA samples. Expected amplicon sizes were as follows; unspliced RNA, 406 bp: E6*I, 224 bp: E6*II, 107 bp. A PCR for GAPDH was performed, to confirm that the RNA was suitable for amplification, using primers that have been previously described [26]. The PCR cycling conditions were as follows; denaturing step of 95 °C for 5 min, followed by 40 cycles of 95 °C for 1 min, 60 °C for 1 min and 72 °C for 1 min. This was followed by a final extension period of 5 min at 72 °C.

### p53 cDNA PCR analysis

p53 amplification was performed on 9 HNSCC samples in which E6/E7 transcripts had been successfully amplified (mean age 63.7 years, 95 % CI 57.41–69.93, range 50–74), together with 12 HNSCC where no E6/E7 transcripts were detectable (mean age 63.2 years, 95 % CI 57.83–68.87, range 46–78). Following unsuccessful amplification of full-length p53 using established primers [27], possibly as a result of RNA fragmentation or degradation, five primer pairs were designed to span the entire open reading frame. PCR was carried out on an Applied Biosystems Veriti using Platinum Taq polymerase (Invitrogen Ltd, UK) and comprised 1x Platinum Taq buffer, 1.5 mM MgCl_2_, 200 μM each dNTP, 200 nM each primer, and 1 U of Platinum Taq polymerase in a 50 μl reaction. The PCR cycling conditions for all primer pairs were as follows; denaturing step of 94 °C for 2 min, followed by 40 cycles of 94 °C for 1 min, 55 °C for 1 min and 72 °C for 1 min. This was followed by a final extension period of 10 min at 72 °C. cDNA generated from primary human foreskin keratinocytes was used as a positive control in this assay.

### Immunohistochemistry analysis

p16^INK4a^ protein is an inhibitor of cyclin dependent kinase and has increased expression with elevated levels of HPV E7. Selected FFPE sections (5 μm) were deparaffinized and antigen target retrieval was performed with citrate-buffer boil (0.1 M Sodium Citrate and 0.1 M Citric acid). Immunohistochemistry was performed as previously described using a mouse monoclonal antibody (BD Biosciences, USA [18].

### Statistical analysis

Statistical calculations were performed using SPSS Version 17 (Chicago, IL, USA). Significance of the association between the detection of HPV16 L1 DNA, HPV16 E6/E7 DNA, HPV16 E6/E7 mRNA, p53 mRNA and P16^INK4a^ was calculated using a two-sided Fisher’s exact test. A Student’s *t*-test was used to compare mean ages. Rates of disease-specific and disease free survival were estimated by means of the Kaplan–Meier method and were compared by the log-rank test. Univariable and multivariable models were developed using Cox regression to investigate size of tumor, nodal status, smoking (never, current or former if smoking cessation >10 years), adjuvant chemoradiotherapy, age, histological differentiation, primary subsite, HPV and p53 status as potential predictors of outcome. Time dependent co-variants were investigated to identify concordance with the proportional hazards assumption.

## Results

### Detection and identification of HPV L1 sequences

Sensitive detection of HPV L1 sequences was performed using a combination of PGMY09/11, Linear Array HPV genotyping test and nested PCR approaches, as previously described [18]. PGMY09/11 PCR detected HPV in 2/42 samples (4.8 %), Linear Array in 10/42 samples (23.8 %) and the nested PCR approach in 29/42 samples (69.0 %). Direct sequencing confirmed the HPV types detected from PGMY09/11 and GP5+/GP6+ nested PCR approaches. Combined results from L1 detection methods showed the presence of HPV 6 in 9/42 (21.4 %) and HPV 16 in 19/42 (45.2 %) samples. Other HPV sequences were detected in 7 samples, but remained unidentifiable due to mixed sequencing traces. HPV 11, HPV 26 and HPV 40 were each found on one occasion in the external meatus, tonsil and oral cavity regions respectively. The distribution of the HPV type 16 can be seen in Table 2. There was no evident association between the detection of HPV 6 or 16 DNA and tumour site.

**Table 2:** Distribution of HPV16 in HNSCCs at different anatomical locations

Anatomical site	HPV16 L1 DNA	Percent	HPV16 E6/E7 DNA	Percent
Larynx (*n* = 16)	6	37.5	8	50
Oropharynx (*n* = 15)	9	60	9	60
Hypopharynx (*n* = 5)	2	40	1	20
Oral cavity (*n* = 4)	2	50	3	75
Other (*n* = 2)	0	0	0	0
Total (*n* = 42)	19	100	21	100

There was no association between the detection of HPV16 DNA and tumour site

### Detection of HPV16 E6/E7 DNA and mRNA

HPV16 E6/E7 DNA was detected in 21/42 samples (50 %). There was a significant association between the detection of HPV16 by E6/E7- and L1-based PCR detection methods (*p* = 0.0016, two-sided Fisher’s exact test). HPV16 E6/E7 transcripts were detected in 9/42 samples (21.4 %). No significant association was seen between the presence of HPV16 L1 DNA and E6/E7 transcript detection (*p* = 1) or the presence of HPV16 E6/E7 DNA and E6/E7 transcript detection (*p* = 1). Of the samples expressing HPV 16 E6/E7, the primary lesion was located in the larynx (x6), hypopharynx (x1), oropharynx (x1) and oral cavity (x1). There was no association between anatomical location and HPV16 E6/E7 expression.

### Clinical evaluation and follow-up

Forty-two patients with primary HNSCC were recruited to this study with a 5-year disease specific survival of 72 % (95 % CI 66–76 %). At the time of surgery, the mean patient age was 62.9 years (range 46–87). The median follow-up time was 53 months (range 45–61 months).

**Fig. 1:**
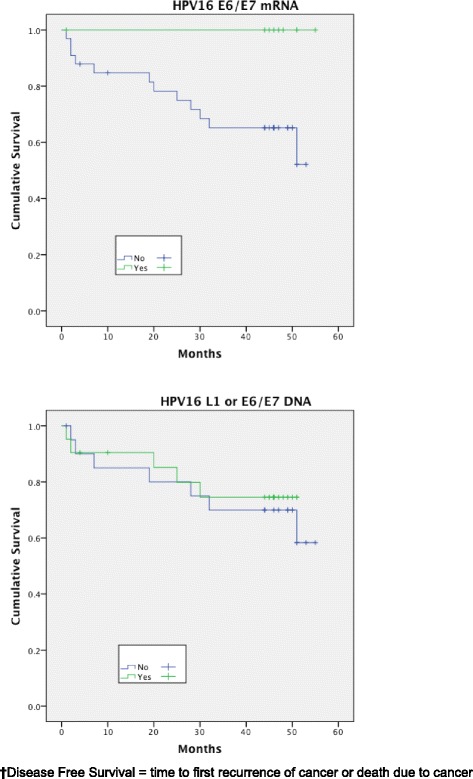
Disease free survival (DFS)^**†**^ stratified by HPV mRNA/DNA (Log- Rank multiple regression analysis *p* = 0.04 and *p* = 0.68, respectively)

**Fig. 2:**
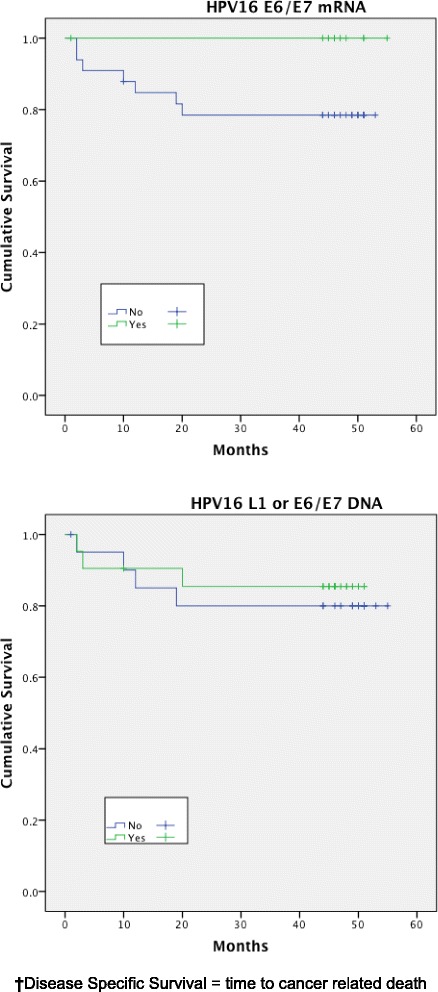
Disease specific survival (DSS)^**†**^ stratified by HPV mRNA/DNA (Log- Rank multiple regression analysis *p* = 0.17 and *p* = 0.66 respectively)

Expression of HPV16 mRNA E6/E7 was found to be a significant predictor of disease free survival on univariable analysis but this was not the case when stratifying by presence of HPV16 L1 or E6/7 DNA (Figs. 1 and 2). Disease specific survival shows the same clear trend but does not quite achieve significance. Multivariable analysis investigated the influence of tumour size (T1-2 versus T3-4), nodal status, smoking, primary surgery, primary radiotherapy, primary surgery + adjuvant radiotherapy, age, histological differentiation (well/moderate/poor SCC) and subsite. Forward model selection confirmed mRNA expression as an independent prognostic factor (Table 3).

**Table 3:** Log-Rank analysis for disease free survival (DFS) and disease specific survival (DSS)

Variable	DFS *p*-value	DSS *p*-value
Node Status (+/−)	0.93	0.22
Primary Site (Larynx/Oropharynx/Hypopharynx/OraL/Other)	0.16	0.12
Tumour size (T1-2 versus T3-4)	0.3	0.45
Smoking (Y/N)	0.68	0.51
Surgery	0.72	0.65
Radiotherapy (RT)	0.19	0.62
Surgery + adjuvant RT	0.34	0.26
Age	0.25	0.79
Histology SCC (Well / Mod / Poor)	0.64	0.78
HPV16 E6/7 mRNA	0.04	0.17
HPV16 L1 or E6/7 DNA	0.68	0.66
P53 (full-length)	0.89	0.17

There was no significant difference in the mean age, irrespective of the method of HPV detection used, between HPV-positive and HPV-negative patients.

### Detection of p53 mRNA

Amplicons spanning the entire open reading frame of p53 were detected in 6/9 (66.7 %) of HPV16 E6/E7 transcript positive HNSCC samples but only in 2/12 (16.7 %) of the age-matched control group, where E6/E7 transcripts were undetectable (Table 4). There was a significant association between E6/E7 expression and the presence of full-length p53 (*p* = 0.032, Fisher’s exact test; OR 10). Full-length p53 expression was not found to correlate with anatomical site, HPV16 L1/E6/E7 DNA or p16^INK4a^.

**Table 4:** p53 detection analysis

Sample ID	Anatomical site	L1 DNA	HPV 16 E6/E7 DNA	HPV 16 mRNA	p53 PCR	Smokers
2	Oropharynx	6	–	–	–	+
5	Larynx	16	–	–	–	–
7	Hypopharynx	–	–	–	–	+
8	Other	–	–	–	–	+
9	Oropharynx	6, 16	–	–	–	+
12	Other	6, 11	–	–	–	–
17	Oropharynx	Mixed	–	–	–	+
18	Larynx	–	–	–	–	+
26	Larynx	6	–	–	+	–
39	Oropharynx	16	–	–	+	–
50	Oropharynx	16	+	–	–	+
52	Hypopharynx	16	+	–	–	–
10	Larynx	6	–	+	–	–
14	Larynx	–	–	+	–	+
15	Larynx	–	+	+	–	–
16	Oral cavity	40	–	+	+	–
33	Larynx	16	+	+	+	+
35	Hypopharynx	16	–	+	+	–
36	Larynx	16	+	+	+	+
41	Oropharynx	16, mixed	+	+	+	–
54	Larynx	–	–	+	+	–

Amplicons spanning the entire p53 open reading frame were amplified from 6/9 HPV 16 E6/E7 mRNA positive HNSCCs (bottom), but only 2/12 transcript negative samples (top) (*p* = 0.032, Fisher’s exact test; OR 10). HPV16 DNA or P16^INK4a^ showed no relationship with p53 PCR

## Discussion

In this study, we report the prevalence of HPV16 DNA and mRNA in 42 unselected HNSCC patients, significantly correlating the presence of HPV16 mRNA expression with the successful amplification of p53 (despite a small sample size). Overall, HPV16 DNA was present in 45.2 % of samples, with detectable E6/E7 mRNA in 21.4 %. In two-thirds of the samples expressing E6/E7, p53 expression was also seen. This study has enabled the classification of HNSCCs into subsets based on both their HPV 16 DNA and mRNA status.

HPV16 and HPV6 were the predominant subtypes detected, being present in 47.6 % and 21.4 % of the samples, respectively. Meta-analysis has revealed that HPV16 is present in the majority of HPV-positive HNSCCs, with HPVs 18, 6 and 11 involved to a lesser extent and an overall prevalence of HPV in HNSCC of 25.9 % ^27^. Large epidemiological studies also suggest that the oropharyngeal site is most associated with high-risk oncogenic HPV subtypes [28, 29].

The higher HPV prevalence observed in this series may be due to the combined use of both sensitive PCR approaches and a commercial assay (Roche Linear Array HPV genotyping test) for the detection of HPV sequences. However, several studies within the meta-analysis found a comparable prevalence, indicating that our findings are within the expected range. There were no cases of HPV 18 positive HNSCC in our study. The predominance of HPV 18 in adenocarcinomas of the cervix indicates a tropism towards glandular tissue [30, 31]. In the context of HNSCC, adenocarcinomas are rare and occur mainly in the salivary glands [32]. No cases of adenocarcinoma were present in this study. The DNA of HPV 26 and HPV 40 were each found on one occasion. HPV 26 has been classified as a probable high-risk type, whereas HPV 40 is a low-risk type [33]. A clear correlation existed between the detection of HPV 16 DNA by L1- and E6/E7-specific PCRs. However, despite 32 samples being concordant, it is noted that 10 samples were positive in one of the PCRs only. This is important for both the estimation of the overall contribution of HPV to HNSCC carcinogenesis and to suggest ‘indirect’ mechanisms by which lesions may arise. The detection of HPV L1 sequences, by Linear Array or PCR amplification, is frequently performed and used as an indicator of HPV-related disease. This has allowed classification of HNSCC tumours into HPV-positive and HPV-negative groups. Several studies have documented significantly better overall outcome in HNSCC patients with tumours positive for HPV DNA compared to their HPV-negative counterparts [9–11, 34].

One of the most significant findings from this study is that p16 positivity does not correlate with HPV E6/E7 mRNA expression. p16^INK4A^ immunohistochemistry outcomes are classified as positive or negative depending on a variable spectrum of detection (ranging from true negative to background staining to false positive to true positive). A systematic review by Grønhøj Larsen et al. [35] identified thirty-nine different studies published between January 1980 to October 2012 which reported sensitivity and specificity of p16 detection (with respect to HPV detection of DNA by PCR). The results ranged from 73 % to 100 % for sensitivity, and 46 % to 100 % for specificity. The definition of “positive” overexpression of p16 is also determined by different % staining cut-off levels such as  ≥ 5 %, ≥5 % but ≤ 70 %, ≥70 %, ≥75 % or even less specific verbal definitions such as “diffuse and strong nuclear cytoplasmic staining”. There are also no standardisations of the anti-p16 monoclonal antibodies used and different clones have different staining avidities and consequently sensitivities and specificities. Our study used a definition of staining ≥70 % of cell cytoplasm and nuclei, seen per field, which stained strongly with the antibody.

This study demonstrates a significant correlation between HPV status and prognosis, but this was restricted to mRNA expression alone. Although this information is important, recent studies suggest further biomarkers may also be required as a subset of the HPV-positive cohort can respond in a manner normally associated with HPV-negative tumors [16, 17]. It is conceivable that HNSCCs positive for L1 but negative for E6/E7 DNA have lost the selective pressure to retain oncogene expression following other genomic events. Conversely, HNSCCs that are E6/E7 positive may have lost L1 sequences, for example following integration, making L1 detection unreliable in determining the role of HPV infection at the time of diagnosis.

The detection of viral oncogene transcripts remains the gold standard by which tumours are classified as HPV positive [36]. In our study, transcript analysis revealed 21.4 % of samples expressed HPV16 E6/E7 mRNA. Although the patients with HPV16 DNA positive tumours tended to be younger (mean age for HPV L1 DNA positive v negative: 60.4 v 64.9 years; mean age for HPV 16 E6/E7 DNA positive v negative: 60.3 v 65.4 years), there was no difference in age between patients with E6/E7 mRNA positive tumours and their mRNA negative counterparts (mean age 63.7 v. 62.6 years). The detection of L1 and E6/E7 DNA in the absence of E6/E7 mRNA expression may reflect virus present as either a passenger or as latent infection whereas the presence of E6/E7 mRNA provides strong molecular evidence for ongoing HPV transcription (and by implication, translation into proteins), which caused or promoted malignant progression in the lesion. HPV-associated cervical carcinomas commonly have integrated HPV genomes in a single copy [37–39]. Therefore, a tumour containing transcriptionally active HPV may well have multiple copies of mature mRNA transcribed from a single copy of HPV DNA. An actively transcribed gene can have mRNA copy numbers several orders of magnitude higher than the DNA from which it originates. This may explain our findings that 4 of the samples were mRNA positive but DNA negative i.e. reflecting insufficiently sensitive DNA detection assays. Sotlar et al. have previously quantified the sensitivity of RT-PCR against DNA-PCR for HPV E6/E7; their data suggest a benefit in favour of the former test with a ten-fold increased detection rate for HPV16 subtype [25].

HPV status, as determined solely by DNA-based detection methods, may be insufficient to predict patient response to treatment and overall outcome. Our study shows that a proportion of HPV DNA positive tumours contain transcriptionally active genomes and this may have more important clinical significance. Other potential prognostic markers, in addition to extensive tobacco and alcohol use, include mutation/over-expression of p53, elevated EGFR levels and over-expression of cyclin D (CCND1) [40, 41]. p53 expression analysis revealed a significant association between HPV 16 E6/E7 expression and the presence of full-length p53. Many studies have revealed inverse correlations between disruptive p53 mutations and HPV in HNSCC [6, 27, 42], mutation of p53 has been shown to result in mis-splicing of transcripts [27, 35, 43] and this may explain the partial amplification seen in the HPV negative samples analysed. However, as a significant number of p53 mutations do not affect full-length expression, this observation may not readily explain all the findings in this study [44]. An alternative method of analysis that may elucidate this in future studies would involve direct sequence analysis of theTP53 gene (exons 5–9) [45]. HPV positive tumours characteristically have wild-type p53; mutations in p53 are not required for tumour development because high-risk HPV E6 targets p53 for degradation [46]. Defects in apoptosis associated with mutated p53 can potentially lead to treatment resistant tumours and may partly explain the improved response seen in HPV associated disease [47].

The five-year clinical observation period for this study, in combination with other studies [48–50], suggests the need to place HPV16 mRNA status as the preferred stratification modality when designing future treatment regimes or clinical trials. This has particular importance when we consider the trend towards “de-intensification” of existing chemotherapeutic regimes in order to decrease treatment related co-morbidity [6, 46, 51].

## Conclusions

In conclusion, the evidence from this small study suggests that HPV status, as determined solely by DNA-based detection methods, is insufficient to predict patient response to treatment and overall outcome in a German population. Our data supports the hypothesis that HPV positive tumours containing transcriptionally active genomes are a more reliable indicator of disease free survival than HPV DNA detection alone. Therefore larger studies are required to establish the frequency of HPV16 mRNA expression in HNSCC. This will determine whether this group, with or without p53 expression, represents a subset of tumours that have improved treatment response.

## Abbreviations

HNSCC, Head & neck squamous cell carcinoma; HPV, human papillomavirus; PCR, polymerase chain reaction
